# Phenotype‐Specific Semi‐Mechanistic Modelling of Florfenicol Time‐Kill Curves in *G. Parasuis* Compared to Other Respiratory Pathogens

**DOI:** 10.1111/jvp.13500

**Published:** 2025-02-07

**Authors:** Andrew Mead, Abigail Hughes, Stefano Azzariti, Pierre‐Louis Toutain, Ludovic Pelligand

**Affiliations:** ^1^ Comparative Biomedical Sciences The Royal Veterinary College London UK; ^2^ INTHERES, Université de Toulouse, INRAE, ENVT Toulouse France; ^3^ Clinical Services and Sciences The Royal Veterinary College London UK

**Keywords:** florfenicol, pharmacodynamics, pharmacokinetics, PK/PD modelling

## Abstract

This study examines the pharmacodynamics (PD) of florfenicol (FFN) for treating porcine respiratory diseases by comparing its effects on *Glaesserella parasuis*, *Actinobacillus pleuropneumoniae* and *Pasteurella multocida* . In vitro time‐kill assays and semi‐mechanistic PD modeling were used to assess bacterial growth and killing rates at varying FFN concentrations. Species‐specific PD models indicated that *f*AUC/MIC was the best PK/PD index across all species. *A. pleuropneumoniae* and *P. multocida* had target values of 1.05 and 1.66 × MIC, respectively for bacteriostasis and 1.12 and 1.87 × MIC for 99.9% kill. Two phenotypes of *G. parasuis* emerged “fast‐kill” and “slow‐kill” which displayed distinct bacterial eradication rates despite similar MICs. For “slow‐kill” isolates, an average free drug concentration (*f*AUC/MIC) of 1.46 and 1.63 × MIC (median, range: 1.53–1.69) was required for bacteriostasis and 99.9% kill. “Fast‐kill” isolates needed an average free drug concentration of 1.38 × MIC for bacteriostasis and 1.51 × MIC for a 99.9% reduction. Indicating that the rate of kill influences the respective average free concentration required to achieve an equivalent antibacterial effect. Simulations of clinical dosing of FFN predicted bacterial eradication for all species, highlighting the value of phenotype‐specific PD modeling in guiding treatment strategies for porcine respiratory infections.

## 1 Introduction

Florfenicol (FFN) is a synthetic antimicrobial drug (AMD), initially derived from chloramphenicol by substitution of the hydroxyl group with fluorine (Ehrlich et al. 1948; Schwarz et al. 2004). FFN inhibits protein synthesis, through binding to the bacterial 50S ribosomal subunit, inhibiting the action of peptidyl transferase (Dowling 2013). Although poorly water soluble, FFN's high lipophilicity results in good tissue penetration (Sams 1995). FFN is a key treatment for porcine respiratory diseases (PRD), a leading cause of morbidity and mortality in swine production (Sargeant et al. 2019) often associated with bacterial infection caused by *Actinobacillus pleuropneumoniae*, *Glaeserella parasuis* (previously *Haemophilus parasuis*) and *Paseteurella multocida* (Opriessnig, Giménez‐Lirola, and Halbur 2011; Register and Brockmeier 2019).

Although the minimum inhibitory concentration (MIC) provides a standardised susceptibility measure to identify if an isolate is susceptible and likely to respond to treatment, it does not provide any information regarding the time‐ or concentration‐dependent killing differences between species, or strains (Toutain et al. 2021). The aim of in vitro time‐kill assays is to investigate the pharmacodynamic (PD) properties of AMDs and determine the bacterial kill rate as it relates to the drug concentration. Quantitative analysis of time‐kill curve (TKC) assays against static drug concentrations offers a standardised and informative way to assess the AMD‐pathogen relationship in relation to simple end‐point susceptibility measurements, such as the MIC (Pelligand et al. 2019). As the TKC establishes the effective rate of killing over a range of AMD concentrations, it is possible to use this data to determine if the extent to which the activity is time‐ or concentration‐dependent (Toutain et al. 2017). In the present investigation, TKC data for susceptible wild‐type isolates of *A. pleuropneumoniae*, *G. parasuis*, and *P. multocida*, from clinical porcine disease cases, have been used to establish the PD parameters using a semi‐mechanistic model (Nielsen and Friberg 2013). These PD parameters (*E*_max_, which represents the maximal drug effect achievable in terms of killing rate; EC_50_, the concentration at which the drug achieves 50% of its maximal effect; and gamma, the Hill coefficient which gives the slope of the concentration‐effect relationship) describe key drug‐response characteristics with gamma for *A. pleuropneumoniae* and *P. multocida* and *E*_max_ for *G. parasuis* being significant in defining the response between these three pathogenic species.

While FFN has been extensively studied in other Pasteurellaceae species, less is known about its detailed PD in *G. parasuis*. The aim of this study was to develop a unifying approach to model TKC of three porcine pathogens and carry out dose fractionation in vitro to identify the key PK/PD indices and their target values for subsequent dose determination. Here we present the novel identification of two distinct bacterial phenotypes within *G. parasuis* populations—designated “fast‐kill” and “slow‐kill”—which show significant differences in their bacterial killing kinetics despite having similar MICs. This phenotypic variability in *G. parasuis* contrasts with the consistent responses observed with *A. pleuropneumoniae* and *P. multocida*. The identification of these two phenotypes highlights the complexity of antimicrobial dynamics within species and underscores the need for tailored therapeutic approaches.

## 2 Materials and Methods

### 2.1 Bacterial Isolates

Isolates of *A. pleuropneumonia* (*n* = 5), *G. parasuis* (*n* = 7), and *P. multocida* (*n* = 5) were provided by ECO Animal Health Ltd. from incidences of porcine disease between 2019 and 2022. The selected isolates are described in Table A1.

Isolates were stored at −80°C in Brain Heart Infusion (BHI; Oxoid, Basingstoke, UK):glycerol (Fisher scientific, Loughborough, UK) prior to analysis and recovered through culture on Mueller‐Hinton (Merck, Steinheim, Germany) fastidious (MH‐F) agar (consisting of MH + 5% mechanically defibrinated horse blood [TCS Bioscience, Botolph Claydon, UK] and 20 mg/L β‐NAD [Glentham Life Science, Corsham, UK]). A minimum of 3 repeated sub‐cultures were performed prior to MIC or TKC analysis in line with the EUCAST guidelines (EUCAST 2022).

### 2.2 Florfenicol

FFN was provided by Produlab Pharma (Forellenweg, Netherlands) with a purity, as supplied (and reported by certificate of analysis), of 100% (< 0.1% loss on drying, ≤ 0.4% impurities). FFN stock solution was prepared, immediately prior to use, at a concentration of 8 mg/mL in 95% ethanol (Fisher scientific, Loughborough, UK). Florfenicol is stable in solution under these conditions, ensuring consistent potency throughout the experimental period (Elimam et al. 2017). Dilution to the working/test concentration was done through direct dilution in either cation‐adjusted Mueller‐Hinton broth (CAMHB; Merck, Steinheim, Germany) or MH‐F broth (CAMHB with added consisting of MH + 5% mechanically defibrinated horse blood [TCS Bioscience, Botolph Claydon, UK] and 20 mg/L β‐NAD [Glentham Life Science, Corsham, UK]) as required.

### 2.3 Minimum Inhibitory Concentration

Minimum inhibitory concentration was determined for each isolate using a twofold dilution series according to the broth microdilution method described in the European Committee for Antimicrobial Testing (EUCAST) guidelines and in accordance with ISO‐20776 (EUCAST 2016). This method was adapted to 5‐overlapping dilution series to increase accuracy to within 20% of the dilution (compared to standard twofold dilution series) as previously described (Mead et al. 2019; Sidhu et al. 2011; Dorey, Hobson, and Lees 2016).

Bacterial suspensions were prepared from individual colonies suspended in PBS with comparison to a 0.5 McFarland standard using DensiCHECK Plus (Biomerieux, Hampshire, UK). Suspension was diluted in CAMHB or MH‐F to achieve final, in‐plate, inoculum of 5 × 10^5^ CFU/mL. The MIC was recorded following overnight static incubation at 37°C in a 5% CO_2_ atmosphere for fastidious organisms. Control isolate *S. pneumoniae* [ATCC 49619 (NCTC 12977 with expected MIC 2 mg/L)] was included in each plate, with MIC accepted within one dilution of the expected target (EUCAST 2020).

### 2.4 Characterisation of Resistance

All isolates were screened for the presence of FFN resistance genes *floR*, *cfr*, *fexA*, *fexB* independently using an adapted polymerase chain reaction (PCR) method described by Li et al. (2020). Briefly, the reaction parameters were as follows: The reaction mixture consisted of 12.5 μL DreamTaq green PCR master mix (Fisher Scientific, UK), 10.5 μL nuclease‐free water (Fisher Scientific, UK), 0.5 μL of each of the 2 primers (10 μM) and 2 μL of DNA template. Thermal lysis of 1 mL of overnight culture at 100°C for 5 min, followed by centrifugation at 17000 × *g*, provided the DNA lysate. The thermal cycler (Techne, UK) conditions were as follows: 5 min denaturation at 94°C, 30 cycles of 40 s at 94°C, 45 s at 55°C, 50 s at 72°C, and a final elongation at 72°C for 10 min.

PCR amplicons were separated using agarose gel electrophoresis (1.5% agarose; Fisher, UK). Amplicon sizes were determined against GeneRuler 100 bp DNA ladder (Fisher Scientific, UK). Controls were included as *floR* positive *E. coli* (NCTC 13846), *fexA* DNA lysate from *S. aureus* (provided by Kees Veldman; Wageningen University), *fexB* positive *E. faecalis* and *cfr* positive *S. hyicus* (provided by Athina Andrea; Technical University of Denmark).

### 2.5 In Vitro Antimicrobial Growth (Time‐Kill) Experiments

TKCs were carried out in triplicate for each isolate for all *G. parasuis* isolates and for a single representative WT of each *A. pleuropneumoniae* (APP_7) and *P. multocida* (PM_6), all other isolates were run once.

The bacterial isolates were cultured overnight on MH or MH‐F agar. Up to 3 colonies were transferred into 3 mL of PBS to achieve density approx. 1 × 10^8^ CFU/mL compared to a 0.5 McFarland standard. This was diluted to achieve an inoculum of approx. 5 × 10^5^ CFU/mL in MH‐F broth and incubated (orbital shaker, 37°C + 5% CO_2_) for 30 min ( *A. pleuropneumoniae* and *P. multocida* ) or 2 h (*G. parasuis*) prior TKC inoculation, to promote optimal log‐phase growth. The final in‐plate inoculum was approx. 5 × 10^5^ CFU/mL and confirmed by viable cell count. Florfenicol was prepared at multiples of MIC, for each respective isolate, and TKC were run in 96‐well plates with each row representing a different concentration (0, 0.25, 0.50, 0.75, 1, 1.5, 2, 3, 4, 6, 8, and 16 × MIC), and each column a different time (0, 1, 2, 4, 8, 12, and 24 h post‐inoculation). Plates were incubated statically at 37°C + 5% CO_2_.

As each well represents an individual sample point (time and MIC multiple) the entire contents (100 μL) were sampled and 10‐fold serially diluted in PBS, covering the estimated range of the bacterial count. The spot‐plate method utilised 10 or 20 μL spots (up to 3 replicate spots) on MH‐F agar, followed by static overnight incubation at 37°C + 5% CO_2_, to allow for colony counts to be performed. Bacterial density (CFU/mL) was then back calculated using the dilution factor and spot volume. Limit‐of‐quantification (LOQ) was 33 or 50 CFU/mL, for *G. parasuis* and *A. pleuropneumoniae* / *P. multocida* respectively.

### 2.6 Pharmacodynamic (PD) Modelling of In Vitro TKC

**FIGURE 1:**
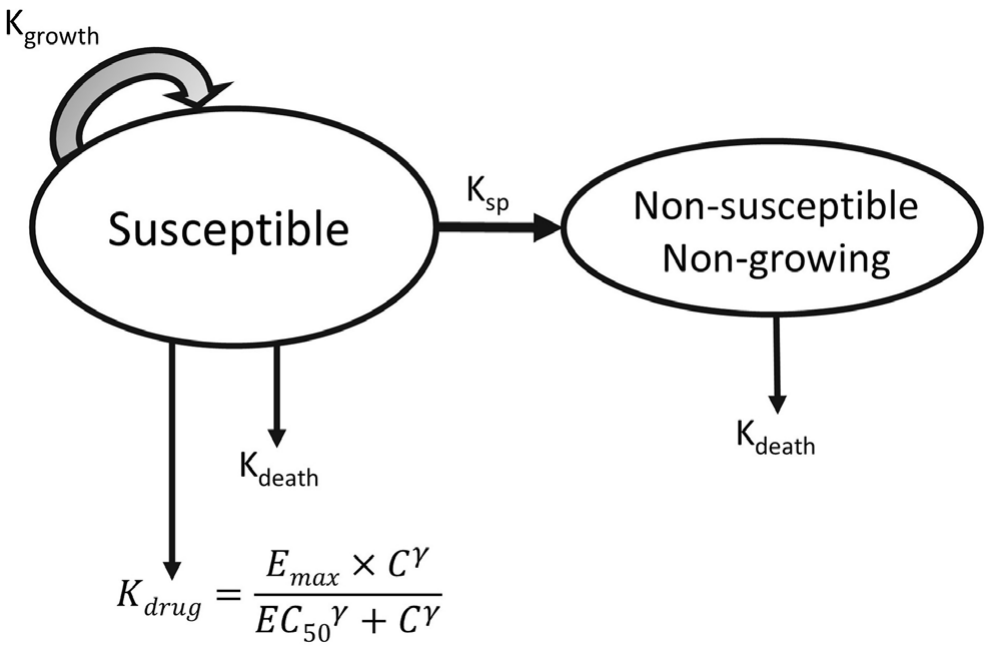
The PK/PD model for FFN time‐kill analysis. All bacteria were assumed to be in a susceptible (S) growing population from initial inoculation, with transfer to a non‐growing and non‐susceptible population. The maximal growth rate (*K*_growth_) applies only to the susceptible population with an irreversible transfer rate to a non‐growing state (*K*_SP_). All bacteria were subject to a constant rate of natural cell death (*K*_death_) fixed to 0.179 h^−1^. The effect of florfenicol (*K*_drug_), having the same dimension as *K*_death_ (h^−1^), was described by a Hill model with three parameters (*E*_max_ for efficacy, EC_50_ for potency and Gamma/γ for the slope), C being the florfenicol concentration at which bacteria are exposed. *K*_drug_ was considered as additive to the natural cell death. To account for isolate variability a random effect was tested on *E*_max_ and gamma. Model details are provided in Appendix B.

Pharmacodynamic data analyses were conducted using Phoenix NLME v8.3.0.5005 (Pharsight Corporation, St Louis, MO, United States). Determination of the PD parameters from time‐kill experiments of all isolates were analysed independently for each species using a semi‐mechanistic PD model adapted from Nielsen and Friberg (2013) as shown in Figure 1.

Values below the limit of quantification (BLQ) were 14.3% (≤ 33 CFU/mL), 20.6% (≤ 49 CFU/mL), and 1.7% (≤ 33 CFU/mL) for *G. parasuis*, *A. pleuropneumoniae* and *P. multocida* , respectively. BLQ data were retained in the analysis by using a likelihood‐based approach according to the M3 method (Beal 2001).

Rival models were compared by the objective function value (OFV) and Akaike information criterion (AIC). Adequacy of model fit was determined through diagnostic goodness‐of‐fit plots including Visual Predictive Check (VPC), obtained by simulating 1000 replicates to plot 20%, 50% and 80% predictive check quantiles with their 90% confidence intervals.

Additional secondary parameters were calculated directly from the model, including MIC as described by Mouton et al. (2018).

### 2.7 In Silico Dose Fractionation

FFN plasma concentration PK data in pigs were obtained from companies manufacturing generic formulations for which PK is compared against reference formulation in bioequivalence studies (Nuflor 300 mg/mL solution). Sixteen male Landrace‐cross 2‐month‐old pigs, dosed with 30 mg/kg intramuscular, sampled pretreatment and at 0.5, 1, 1.5, 2, 2.5, 3, 4, 5, 6, 8,12, 24, 30, 48, and 72‐h post‐injection. A simple one‐compartment model was used to fit this PK data. This was combined with the best fitting PD model for each species which included a random effect on *E*_max_ (*G. parasuis*) or gamma (*A. pleuropneumoniae, P. multocida*). The resulting PK/PD model was used to simulate the free plasma florfenicol concentration (Cfree) profile, as adjusted for 15% protein binding (Lei et al. 2018), and the bacterial populations (S, P, and total) over 24 h. Doses of 0, 1.3, 2.4, 4.6, 6.8, 9.0, 11.2, and 20 mg/kg (pre‐protein binding) as a single administration, half‐dose administered every 12 h, or quarter‐dose administered every 6 h were simulated, giving a total of 24 possible exposure patterns. A wide‐range of exposure patterns ensures robust evaluation of the relationship between drug exposure and antibacterial effect.

Dose fractionation was performed for each isolate, for each species, separately by using the EC_50_ parameter estimates for the individual isolate, the population parameter estimates for growth rate (*K*_growth_), natural death rate (*K*_drug_), maximal bacterial density (*B*_max_), and the posthoc values for maximum drug effect (*E*_max_) for *G. parasuis* or potency (gamma) for *A. pleuropneumoniae* and *P. multocida* . PK/PD indices: *f*AUC/MIC (area under the Cfree curve divided by predicted MIC over 24 h) and *f*%T>MIC (percentage time Cfree exceeds predicted MIC in 24 h) for each isolate were secondarily calculated from the simulation along with natural log of bacterial density at 24 h (Ln CFU/mL_24h_).

Regression analysis (*I*_MAX_ model) was used to fit dependent variables: *f*AUC/MIC and *f*%T>MIC against the independent variable Ln CFU/mL_24h_. The Akaike Information Criterion (AIC), the weighted sum of the square of the residuals (WSSR), and visual inspection of graphs were used to select the best fitting PK/PD index for each isolate.

Target values for bacteriostasis, as defined as achieving a final bacterial load of 1 × 10^5^ CFU/mL at 24 h equivalent to the initial target inoculum, and a 99.9% (or 3log_10_) reduction from bacteriostasis (i.e., a bacterial load at 24 h of 1 × 10^2^ CFU/mL) were determined by solving the equation obtained for the best fit of the I_MAX_ model. *f*AUC/MIC was converted to an 24 h average hourly Cfree (as described by Toutain, Bousquet‐Melou, and Martinez 2007).

### 2.8 Dose‐Effect Forecasting

Simulations were performed to forecast the effect of florfenicol treatment, for a single or 2 doses of 15 mg/kg repeated at a 48‐h interval. PD parameters were for representative strains of each species, *G. parasuis* (GP_9; “slow‐kill” and GP_10; “fast‐kill”), *A. pleuropneumoniae* (APP_7), and *P. multocida* (PM_6).

## 3 Results

### 3.1 Minimal Inhibitory Concentration (MIC)

MIC by 2‐fold dilution and 5‐overlapping 2‐fold dilutions for each isolate, across the three bacterial species is shown in Table 1. The MIC ranges for *A. pleuropneumoniae* were 0.45–0.60 mg/L, *G. parasuis* were 0.25–0.40 mg/L, for *P. multocida* were 0.12–0.35 mg/L. All isolates of *A. pleuropneumoniae* and *P. multocida* were susceptible as defined by MICs below the ECOFF (1 mg/L; EUCAST (accessed Oct 2024)). *G. parasuis* isolates were presumed susceptible as their MIC did not exceed the mode of aggregated distribution in the EUCAST database (no ECOFF available or breakpoint available for *G. parasuis*).

**TABLE 1:** Susceptibility as determined by the MIC (mg/L) using standard 2‐fold serial dilution and 5‐overlapping 2‐fold serial dilution.

Isolate identification number	Minimum inhibitory concentration (mg/L)		Susceptibility (susceptible/resistant)^a^
	2‐fold dilution	5‐overlapping dilution	
*A. pleuropneumoniae*			
APP_6	0.5	0.4	Susceptible
APP_7	1	0.6	Susceptible
APP_8	1	0.6	Susceptible
APP_9	0.5	0.45	Susceptible
APP_10	1	0.6	Susceptible
*G. parasuis*			
GP_2	0.5	0.25	Susceptible
GP_3	0.5	0.3	Susceptible
GP_4	0.5	0.25	Susceptible
GP_5	0.5	0.4	Susceptible
GP_6	0.5	0.5	Susceptible
GP_9	0.5	0.3	Susceptible
GP_10	0.5	0.3	Susceptible
*P. multocida*			
PM_6	0.5	0.35	Susceptible
PM_7	0.5	0.35	Susceptible
PM_8	0.5	0.35	Susceptible
PM_9	0.25	0.125	Susceptible
PM_10	0.25	0.115	Susceptible

*Note:* https://mic.eucast.org/search/?search%5Bmethod%5D=mic&search%5Bantibiotic%5D=95&search%5Bspecies%5D=‐1&search%5Bdisk_content%5D=‐1&search%5Blimit%5D=50.

^a^ Susceptibility (wild‐type) is defined as MIC≤ECOFF. EUCAST ECOFF for florfenicol at time of writing were *A. pleuropneumoniae* = 1 mg/L, *P. multocida* = 1 mg/L, *G. parasuis* = No defined ECOFF.

All isolates were screened for the presence of FFN resistance genes *floR*, *cfr*, *fexA* and *fexB*. None of the resistant genes tested were identified among the isolates in this study.

### 3.2 Time‐Kill Assays (TKC)

**FIGURE 2:**
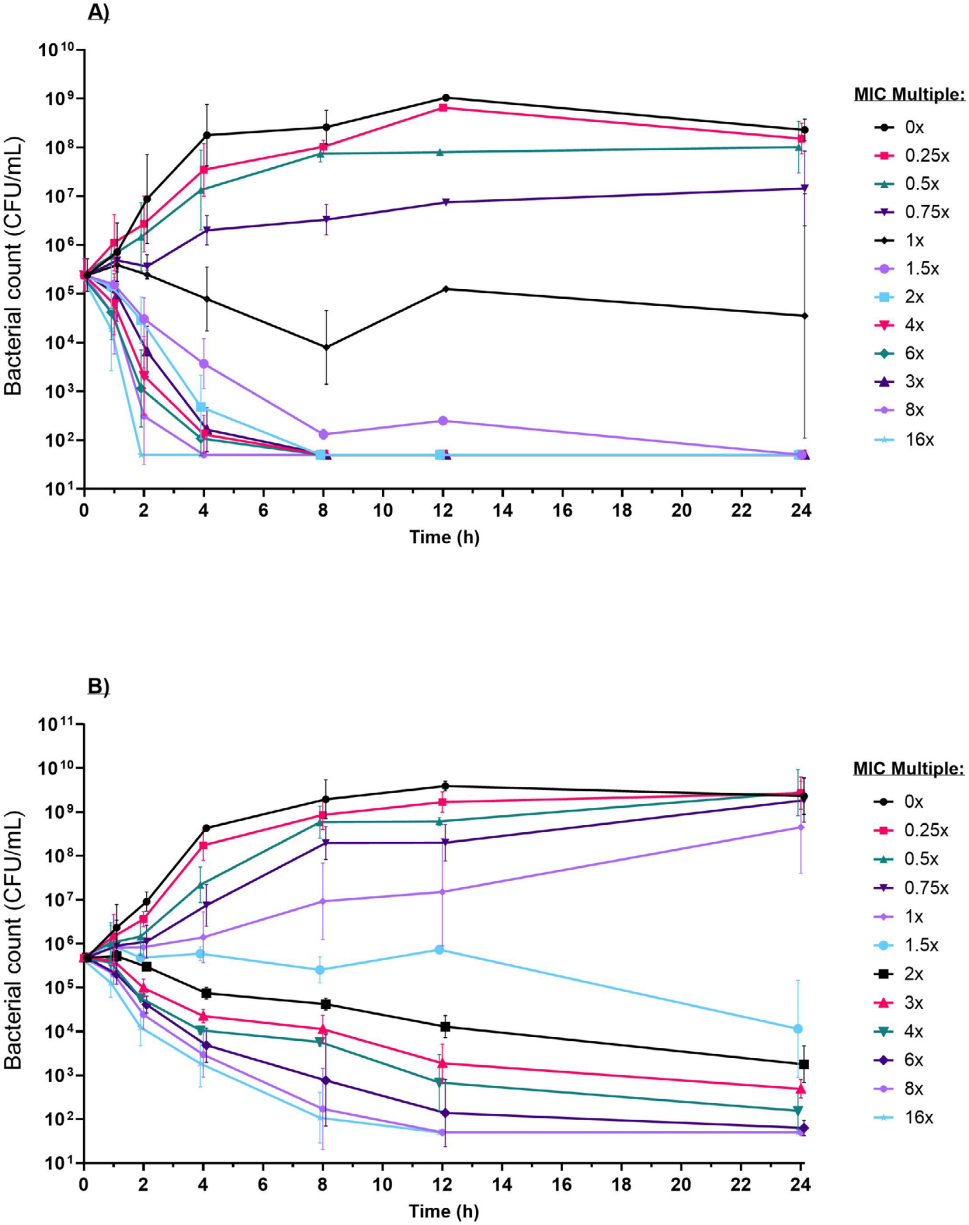
Time‐kill curve (TKC) assay for (A) *A. pleuropneumoniae* 7 (MIC = 0.6 mg/L) and (B) *P. multocida* 6 (MIC = 0.35 mg/L); geometric mean of replicates (with SD) at an initial target inoculum of 5 × 10^5^ CFU/mL at multiples (0, 0.25, 0.5, 0.75, 1, 1.5, 2, 4, 6, 8, and 16) of MIC. LOQ = 50 CFU/mL.

**FIGURE 3:**
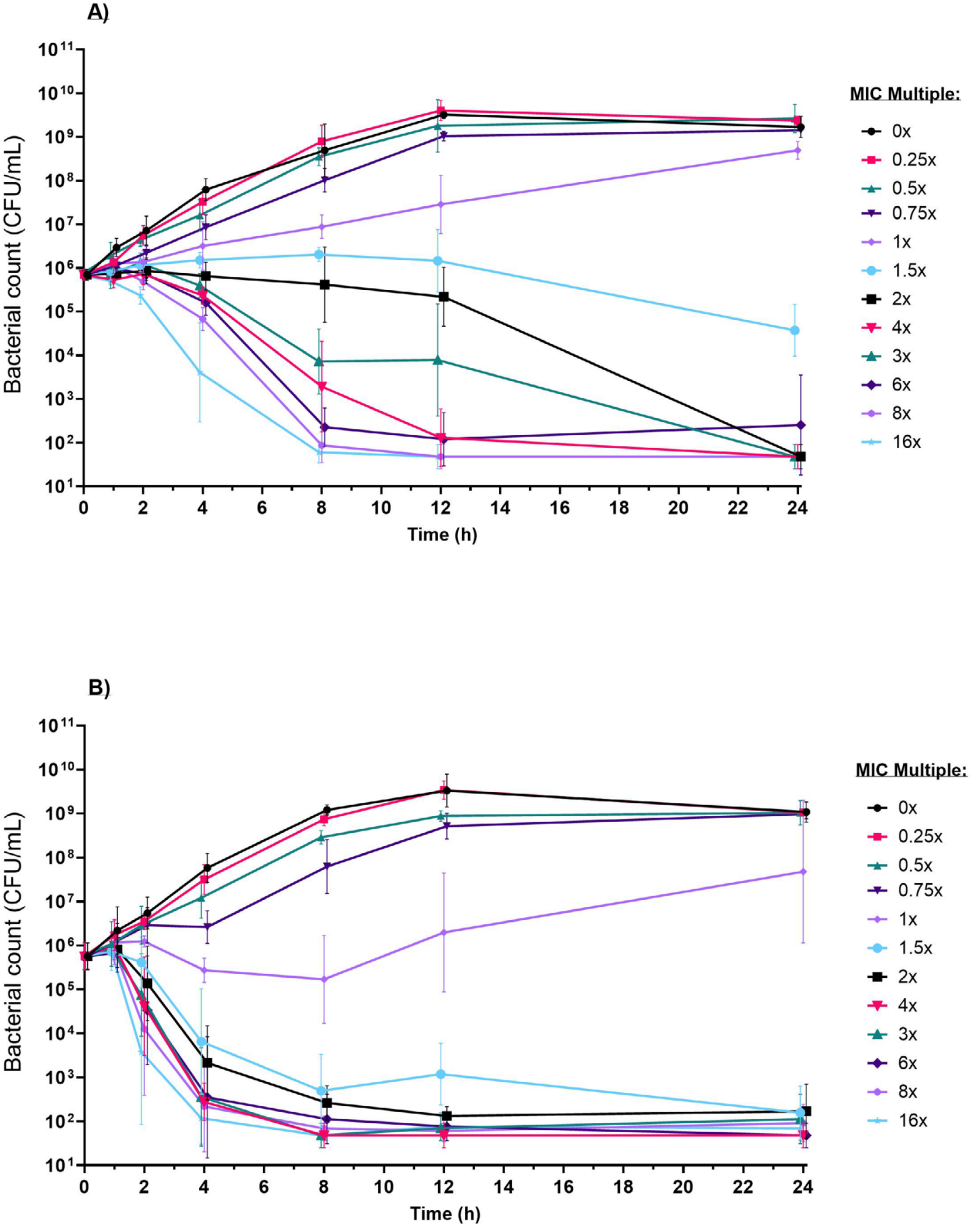
Time‐kill curve (TKC) assay for (A) *G. parasuis* 9 (MIC = 0.3 mg/L; slow‐kill), and (B) *G. parasuis* 10 (MIC = 0.3 mg/L; fast‐kill); geometric mean of replicates (with SD) at an initial target inoculum of 5 × 10^5^ CFU/mL at multiples (0, 0.25, 0.5, 0.75, 1, 1.5, 2, 4, 6, 8 and 16) of MIC. LOQ = 33 CFU/mL.

For representative wild‐type susceptible strains the geometric mean of three replicates is shown for APP_7 and PM_6 in Figure 2 and GP_9 and GP_10 in Figure 3 (individual replicates for all isolates are included in Data S1). Visual inspection of the curves showed that all strains were capable of exponential growth in the absence of florfenicol with a negligible lag‐phase.

*A. pleuropneumoniae* reached a maximal bacterial population (stationary phase) at approx. 10^8^ CFU/mL (Figure 2A) from 8 h with a geometric mean density of 2.60 × 10^8^ CFU/mL (range: 1.5 × 10^8^–6.5 × 10^8^). A rapid bactericidal (≥ 3 log reduction) effect was typically observed for susceptible/wild‐type isolates, with bacterial kill achieved when the FFN concentration exceeds 1–1.5 × MIC, and often resulting in eradication. Comparatively, the rate of kill with concentrations exceeding the MIC was higher with *A. pleuropneumoniae* than with either *P. multocida* or *G. parasuis*, as observed by the increased gradient (i.e., increased rate of kill over time) of the killing curves.

*P. multocida* reached a maximal bacterial population (stationary phase) at approx. 10^9^ CFU/mL (Figure 2B) from 8 h with a geometric mean density of 1.94 **×** 10^9^ CFU/mL (range: 0.60 **×** 10^9^–3.5 **×** 10^9^). Bacterial retardation of growth/kill showed a graduated concentration dependent relationship at concentrations both above and below the MIC.

*G. parasuis* reached a maximal bacterial population (stationary phase), consistent across all isolates, by 12 h at approx. 5.43 × 10^9^ CFU/mL (range: 4.15 × 10^9^–7.10 × 10^9^ CFU/mL). Two phenotypes were identified, differing by speed‐of‐kill at the highest FFN concentration even when expressing the same MIC, are described here as “slow‐kill” and “fast‐kill”. Slow‐kill isolates (GP_3, GP_4, GP_6, GP_9) reached eradication by 8 h (Figure 3A) and fast‐kill isolates (GP_2, GP_5, GP_10) reached eradication by 4 h (Figure 3B). When assuming a linear kill rate between 0 h and eradication (4 or 8 h) these two groups were statistically identifiable (*t*‐test, *p* < 0.01). Visual inspection of the TKC revealed a concentration‐dependent drug effect; the rate of kill increased with florfenicol concentration for all isolates. Eradication (at least 3× log 10 reduction) was achieved in all isolates at florfenicol concentrations ≥ 2× MIC by 24 h, except GP_3 which achieved a 2.81 × log_10_ reduction at 2× MIC and eradication to below LOQ at ≥ 4× MIC.

### 3.3 PD Modelling of Time‐Kill Experiments

#### 3.3.1 A. pleuropneumoniae

The optimal PD model for *A. pleuropneumoniae* shared growth parameters (maximal growth rate; *K*_growth_ and maximum bacterial density; *B*_max_) across all isolates. Results presented in Table 2. Maximal count *B*_max_ was estimated as 6.02 × 10^8^ CFU/mL. The growth rate constant *K*_growth_ was estimated to 1.95 h^−1^ (mean generation time of 31 min). PD parameters separated the individual isolates with a common *E*_max_, an independent estimation of EC_50_ coded as an additive difference from the reference isolate APP_7, and a random effect on gamma. Post hoc (empirical Bayesian) parameter estimates were obtained for each isolate including the EC_50_ and gamma deviations from the population values. Model estimated MICs show correlation with measured MICs as shown in Table 2.

**TABLE 2:** Typical value (tv), median parameter estimates, and 95% confidence intervals of the semi‐mechanistic model describing TKC for *A. pleuropneumoniae.* Measured MIC and model estimated MIC are shown for each isolate and are in‐line with the estimated EC_50_.

		Estimated parameters				Computed MIC (see Mouton and Vink)^c^		
Parameters^a^	Unit	Typical value (tv) estimate	Median (Bootstrap) estimate^b^	Confidence interval (from Bootstrap)		MIC; mg/L (as calculated from the model^c^)	MIC; mg/L (as measured by 5‐overlapping dilutions)	MIC ratio (calculated: measured)
				2.5% CI	97.5% CI			
Bacterial growth system parameters								
*K*_growth_	h^−1^	1.949	1.943	1.622	2.236			
*K*_death_	h^−1^	0.179 (fixed)	—	—	—			
*B*_max_	CFU/mL	6.02 × 10^8^	5.88 × 10^8^	2.21 × 10^8^	5.36 × 10^9^			
Pharmacodynamic parameters								
EC_50_ (APP_6)	mg/L	0.515	0.450	0.225	0.713	0.26	0.40	0.66
EC_50_ (APP_7)	mg/L	0.637	0.623	0.477	0.721	0.51	0.60	0.85
EC_50_ (APP_8)	mg/L	0.647	0.612	0.394	0.787	0.44	0.60	0.73
EC_50_ (APP_9)	mg/L	0.869	0.559	0.340	1.159	0.48	0.45	1.07
EC_50_ (APP_10)	mg/L	0.689	0.592	0.377	0.862	0.44	0.50	0.87
Gamma	scalar	0.968	1.099	0.836	1.854			
*E*_max_	h^−1^	3.907	3.837	2.309	4.602			

^a^ *K*_*growth*_, maximal growth rate constant; *K*_death_, natural death rate; *B*_max_, maximum possible bacterial density; EC_50_, concentration required to achieve 50% of *E*_max_ as calculated for each strain; γ/gamma, Hill coefficient; *E*_max_, maximal increase in kill effect in addition to *K*_death_.

^b^ Bootstrap estimates, *n* = 50.

^c^ MIC, minimum inhibitory concentration calculated based on the equation from Mouton and Vinks (2005) with a standard inoculum of 5 × 10^5^  CFU/mL.

**FIGURE 4:**
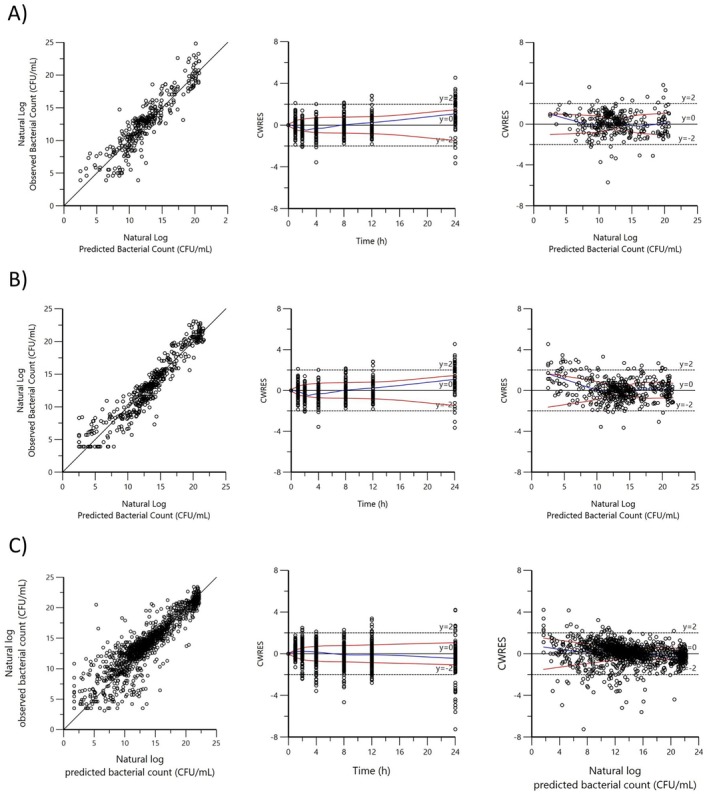
Goodness of fit plots for (A) *A. pleuropneumoniae* , (B) *P. multocida* , and (C) *G. parasuis*: Observed bacterial count vs. predicted bacterial count; Conditional weighted residuals (CWRES) versus Time (h) (IVAR); Conditional weighted residuals vs. predicted bacterial count (CWRES vs. PRED). CWRES are calculated as the difference (approximated by first‐order conditional estimation) between the observed and predicted values divided by the root of the covariance of the observed data. These should fall within 2 standard deviations (*y* = −2 to *y* = 2, dotted lines) of 0. All bacterial counts are plotted as natural logarithms of the CFU/mL.

**FIGURE C1:**
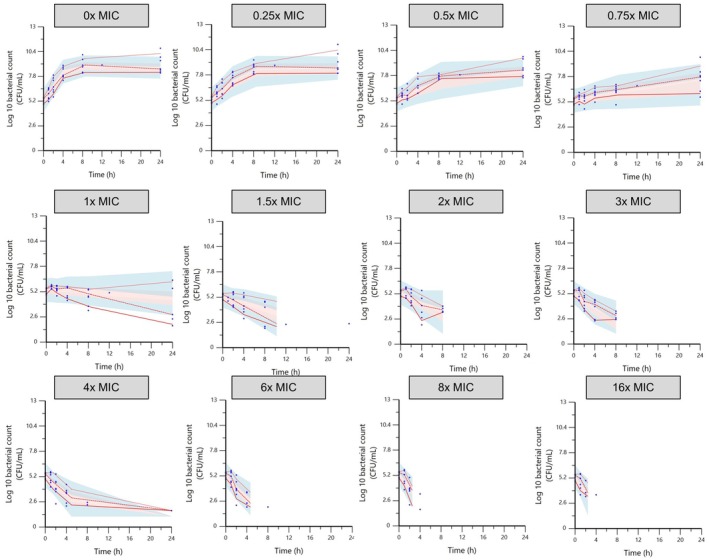
Visual Predictive Check (VPC) for five *A. pleuropneumoniae* isolates obtained with simulation of 1000 replicates. The observed quantiles (20%, 50% and 80%) (red lines) are reasonably well super‐imposed for most TKC with the corresponding 90% confidence interval (shaded area) of simulated data. Upper and lower blue shaded area are the 90% CI of quantile 20% and 80% respectively and the pink shaded area is the 90% CI of quantile 50%, Blue circles: Observed data.

Goodness‐of‐fit plots are shown in Figure 4A and Visual predictive check (VPC) in Figure C1.

#### 3.3.2 P. multocida

The optimal PD model for *P. multocida* shared growth parameters (maximal growth rate; *K*_growth_ and maximum bacterial density; *B*_max_) across all isolates. Results presented in Table 4. Maximal count *B*_max_ was estimated as 1.66 × 10^9^ CFU/mL. The growth rate constant *K*_growth_ was estimated to 2.14 h^−1^ (mean generation time of 28 min). PD parameters separated the individual isolates with a common *E*_max_, an independent estimation of EC_50_ coded as an additive difference from the reference isolate PM_6, and a random effect on gamma. Post hoc (empirical Bayesian) parameter estimates were obtained for each isolate including the EC_50_ and gamma deviations from the population values. Model estimated MICs show correlation with measured MICs as shown in Table 3.

**TABLE 3:** Typical value (tv), median parameter estimates, and 95% confidence intervals of the semi‐mechanistic model describing TKC for *P. multocida.* Measured MIC and model estimated MIC are shown for each isolate and are in‐line with the estimated EC_50_.

Parameters^a^	Unit	Typical value (tv) estimate	Median (Bootstrap) estimate^b^	Confidence interval (from Bootstrap)		MIC; mg/L (as calculated from the model^c^)	MIC; mg/L (as measured by 5‐overlapping dilutions)	MIC ratio (calculated: measured)
				2.5% CI	97.5% CI			
Bacterial growth system parameters								
*K*_growthmax_	h^−1^	2.140	2.132	1.936	2.620			
*K*_death_	h^−1^	0.179 (fixed)	—	—	—			
*B*_max_	CFU/mL	1.66 × 10^9^	1.66 × 10^9^	1.17 × 10^9^	2.08 × 10^9^			
Pharmacodynamic parameters								
EC_50_ (PM_6)	mg/L	0.339	0.342	0.295	0.608	0.38	0.35	1.10
EC_50_ (PM_7)	mg/L	0.215	0.266	0.154	0.608	0.27	0.35	0.76
EC_50_ (PM_8)	mg/L	0.435	0.413	0.288	1.067	0.51	0.35	1.46
EC_50_ (PM_9)	mg/L	0.158	0.143	0.087	0.610	0.20	0.13	1.59
EC_50_ (PM_10)	mg/L	0.107	0.108	0.060	0.226	0.12	0.12	1.06
Gamma	scalar	0.782	0.792	0.471	1.016			
*E*_max_	h^−1^	3.270	3.224	2.742	5.303			

^a^ *K*_growth_, maximal growth rate constant; *K*_death_, natural death rate; *B*_max_, maximum possible bacterial density; EC_50_, concentration required to achieve 50% of *E*_max_ as calculated for each strain; γ/gamma, Hill coefficient; *E*_max_, maximal increase in kill effect in addition to *K*_death_.

^b^ Bootstrap estimates, *n* = 50.

^c^ MIC, minimum inhibitory concentration calculated based on the equation from Mouton and Vinks (2005) with a standard inoculum of 5 × 10^5^ CFU/mL.

**FIGURE C2:**
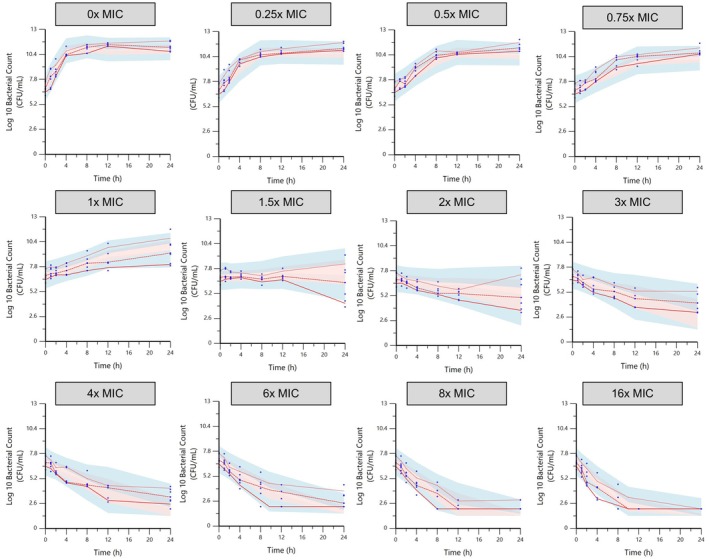
Visual Predictive Check (VPC) for five susceptible *P. multocida* isolates obtained with simulation of 1000 replicates. The observed quantiles (20%, 50% and 80%) (red lines) are reasonably well super‐imposed for most TKC with the corresponding 90% confidence interval (shaded area) of simulated data. Upper and lower blue shaded area are the 90% CI of quantile 20 and 80% respectively and the pink shaded area is the 90% CI of quantile 50%, Blue circles: Observed data.

Goodness‐of‐fit plots are shown in Figure 4B and Visual predictive check (VPC) in Figure C2.

#### 3.3.3 G. Parasuis

The optimal PD model for *G. parasuis* shared growth parameters (maximal growth rate; *K*_growth_ and maximum bacterial density; *B*_max_) across all isolates irrespective of proposed phenotype. Results presented in Table 4. Maximal count *B*_max_ was estimated as 2.8 × 10^9^ CFU/mL. The growth rate constant *K*_growth_ was estimated to 1.17 h^−1^ (mean generation time of 51 min). PD parameters separated the individual isolates with a common gamma, an independent estimation of EC_50_ as an additive difference from the reference isolate GP_9, and a random effect on *E*_max_. *Post hoc* (empirical Bayesian) parameter estimates were obtained for each isolate including the EC_50_ and *E*_max_ deviations from the population values. Model estimated MICs show correlation with measured MICs as shown in Table 4.

**TABLE 4:** Typical value (tv), median parameter estimates, and 95% confidence intervals of the semi‐mechanistic model describing TKC for *G. parasuis*. Measured MIC and model estimated MIC are shown for each isolate and are in‐line with the estimated EC_50_.

Parameters^a^	Unit	Typical value (tv) estimate	Median (bootstrap) estimate^b^	Confidence interval (from bootstrap)		MIC; mg/L (as calculated from the model^c^)	MIC; mg/L (as measured)	MIC ratio (calculated: measured)
				2.5% CI	97.5% CI			
Bacterial growth system parameters								
*K*_growthmax_	h^−1^	1.171	1.176	1.114	1.251			
*K*_death_	h^−1^	0.179 (fixed)	—	—	—			
*B*_max_	CFU/mL	2.8 × 10^9^	2.7 × 10^9^	1.6 × 10^9^	4.4 × 10^9^			
Pharmacodynamic parameters								
EC_50_ (GP_2)	mg/L	0.541	0.569	0.387	0.833	0.30	0.25	1.20
EC_50_ (GP_3)	mg/L	0.715	0.685	0.489	0.942	0.41	0.30	1.37
EC_50_ (GP_4)	mg/L	0.335	0.408	0.301	0.688	0.25	0.25	1.00
EC_50_ (GP_5)	mg/L	0.895	0.866	0.549	1.294	0.49	0.40	1.23
EC_50_ (GP_6)	mg/L	0.656	0.693	0.601	0.820	0.47	0.50	0.94
EC_50_ (GP_9)	mg/L	0.562	0.541	0.440	0.748	0.35	0.30	1.16
EC_50_ (GP_10)	mg/L	0.562	0.574	0.437	0.850	0.26	0.30	0.87
Gamma	scalar	1.528	1.560	1.237	2.130			
*E*_max_	h^−1^	2.485	2.546	2.231	3.017			

^a^ *K*_growth_, maximal growth rate constant; *K*_death_, natural death rate; *B*_max_, maximum possible bacterial density; EC_50_, concentration required to achieve 50% of *E*_max_ as calculated for each strain; γ/ gamma (Hill coefficient), slope of concentration effect curve; *E*_max_, maximal increase in kill effect in addition to *K*_death_.

^b^ Bootstrap estimates, *n* = 100.

^c^ MIC, minimum inhibitory concentration calculated based on the equation from Mouton and Vinks (2005) with a standard inoculum of 5 × 10^5^ CFU/mL.

**FIGURE C3:**
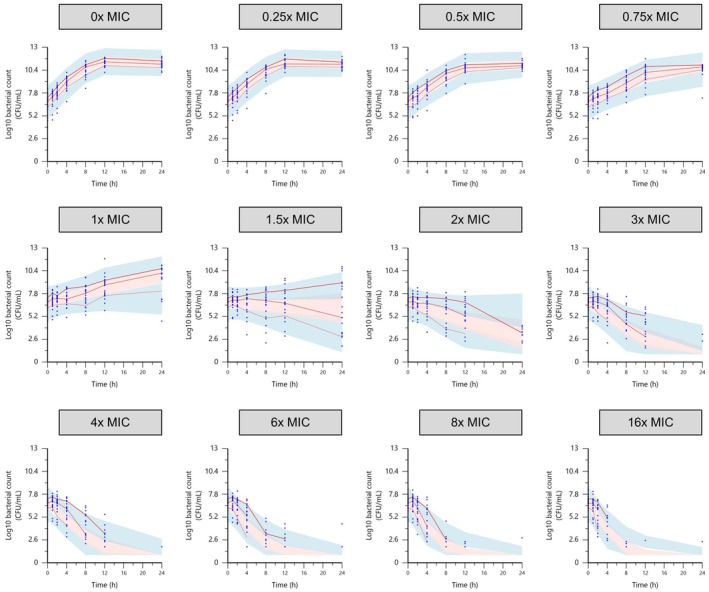
Visual Predictive Check (VPC) for seven *G. parasuis* isolates obtained with simulation of 1000 replicates. The observed quantiles (20%, 50%, and 80%) (red lines) are reasonably well super‐imposed for most TKC with the corresponding 90% confidence interval (shaded area) of simulated data. Upper and lower blue shaded area are the 90% CI of quantile 20 and 80% respectively and the pink shaded area is the 90% CI of quantile 50%, Blue circles: Observed data.

Goodness‐of‐fit plots are shown in Figure 4C and Visual predictive check (VPC) in Figure C3.

### 3.4 PK/PD Index Determination by Dose Fractionation

#### 3.4.1 *A. pleuropneumoniae* and *P. multocida*

**FIGURE 5:**
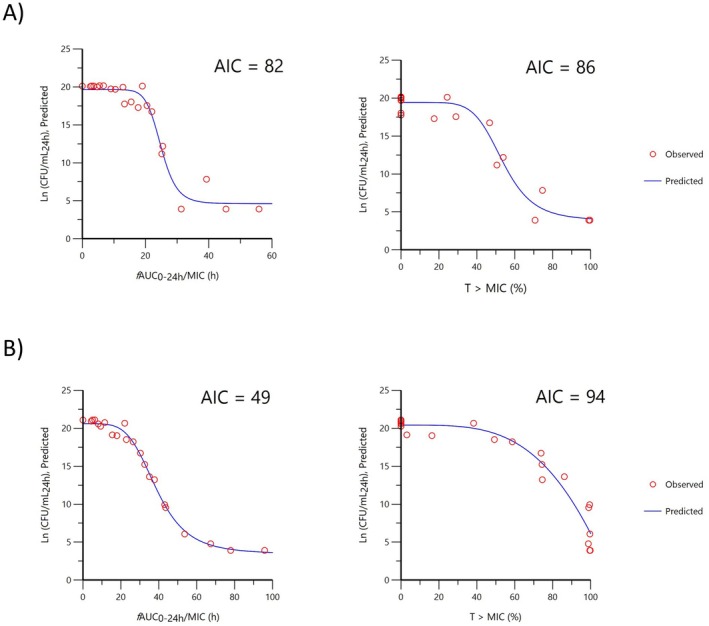
Comparison of fitting for (A) *A. pleuropneumoniae* and (B) *P. multocida* of predicted natural log of bacterial density at 24 h (Ln CFU/mL_24h_) for *f*AUC/MIC (area under the Cfree curve divided by predicted MIC over 24 h) or with *f*%T>MIC (percentage time Cfree exceeds predicted MIC in 24 h). A sigmoid I_MAX_ model was used. A lower AIC indicates a better model.

In silico dose fractionation using a combined IV PK/PD model demonstrated that for a typical isolate of *A. pleuropneumoniae* (Figure 5A) and *P. multocida* (Figure 5B), the PK/PD index *f*AUC/MIC (AIC = 82 and 49, respectively) predicted outcomes in the Imax model more accurately than *f*%T_0–24h_ > MIC (AIC = 86 and 94, respectively).

Bacteriostasis and a 99.9% (or 3log_10_) reduction from bacteriostasis were achieved for *A. pleuropneumoniae* when the average Cfree over 24 h exceeded 1.05 × MIC, and 1.12 × MIC, respectively. For *P. multocida* the 24‐h average Cfree to achieve either bacteriostasis or a 99.9% reduction from stasis were 1.66 × MIC and 1.87 × MIC respectively (24‐h average Cfree calculated from *f*AUC/MIC_24_), (Table 5).

**TABLE 5:** *f*AUC_24_/MIC to achieve bacteriostasis or 90% reduction from stasis for typical isolates of *A. pleuropneumoniae* and *P. multocida* .

Isolate	*f*AUC/MIC_24_ for Bacteriostasis (h)	24‐h Average Cfree/MIC for Bacteriostasis (scalar)	*f*AUC/MIC_24_ for 99.9% reduction from stasis (h)	24‐hAverage Cfree/MIC for 99.9% reduction from stasis (scalar)
*A. pleuropneumoniae*	25.3	1.054	26.9	1.121
*P. multocida*	39.7	1.656	44.8	1.867

**FIGURE 6:**
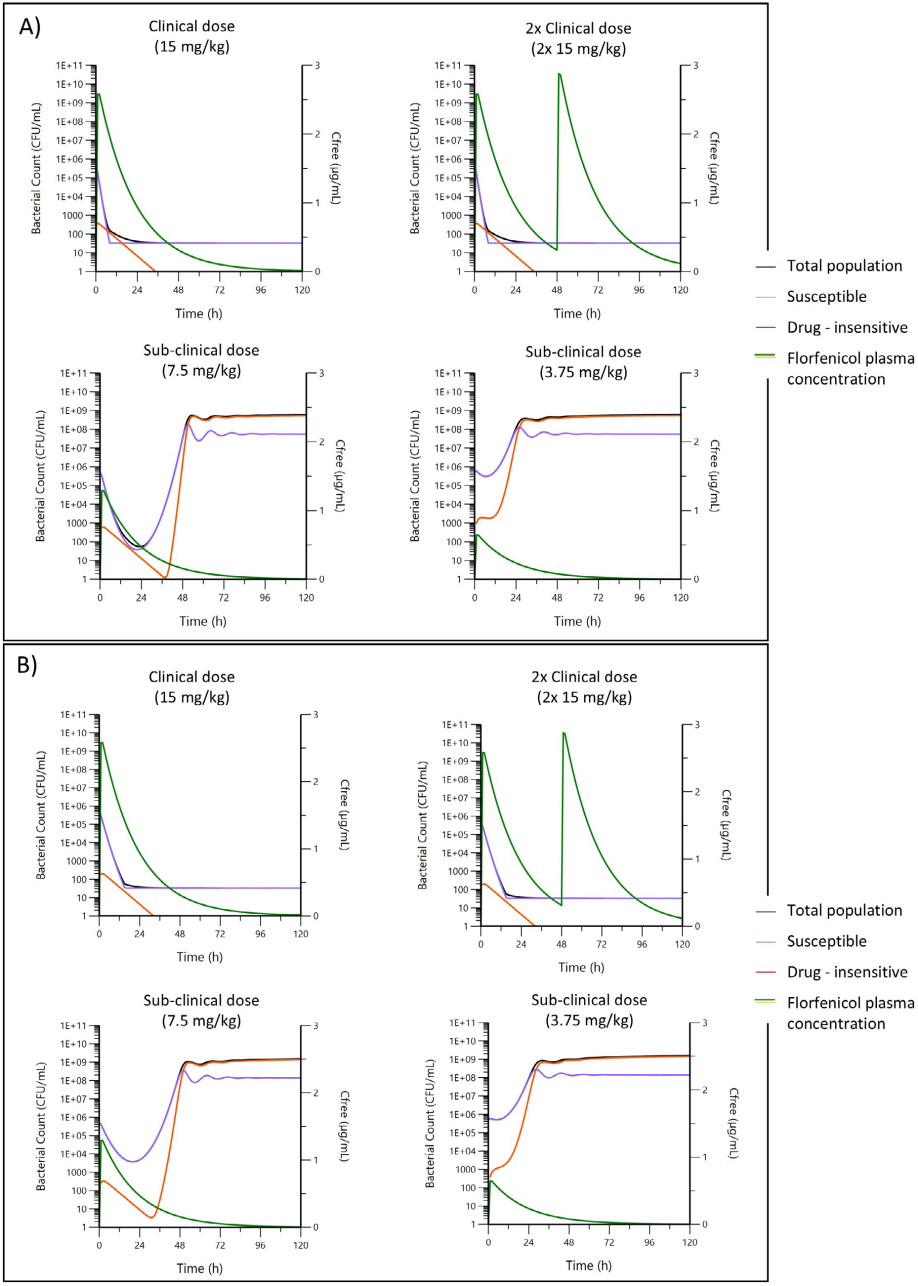
Simulated PK/PD profiles for a typical strain of (A) *A. pleuropneumoniae* and (B) *P. multocida* with an equivalent Nuflor formulation, free concentration (μg/mL; green line) adjusted for 15% protein binding, at the clinical dose once, a repeated clinical dose after 48 h, or at sub‐clinical half and quarter doses. Initial bacterial load of 5 × 10^5^ CFU/mL and predicted bacterial response for both the susceptible (blue line), persistent drug insensitive (red line), and total (black line) populations.

PK/PD simulation for a 15 mg/kg standard formulation forecasted eradication of both *A. pleuropneumoniae* (Figure 6A) and *P. multocida* (Figure 6B), while a sub‐clinical dose failed to achieve eradication.

#### 3.4.2 G. Parasuis

**FIGURE 7:**
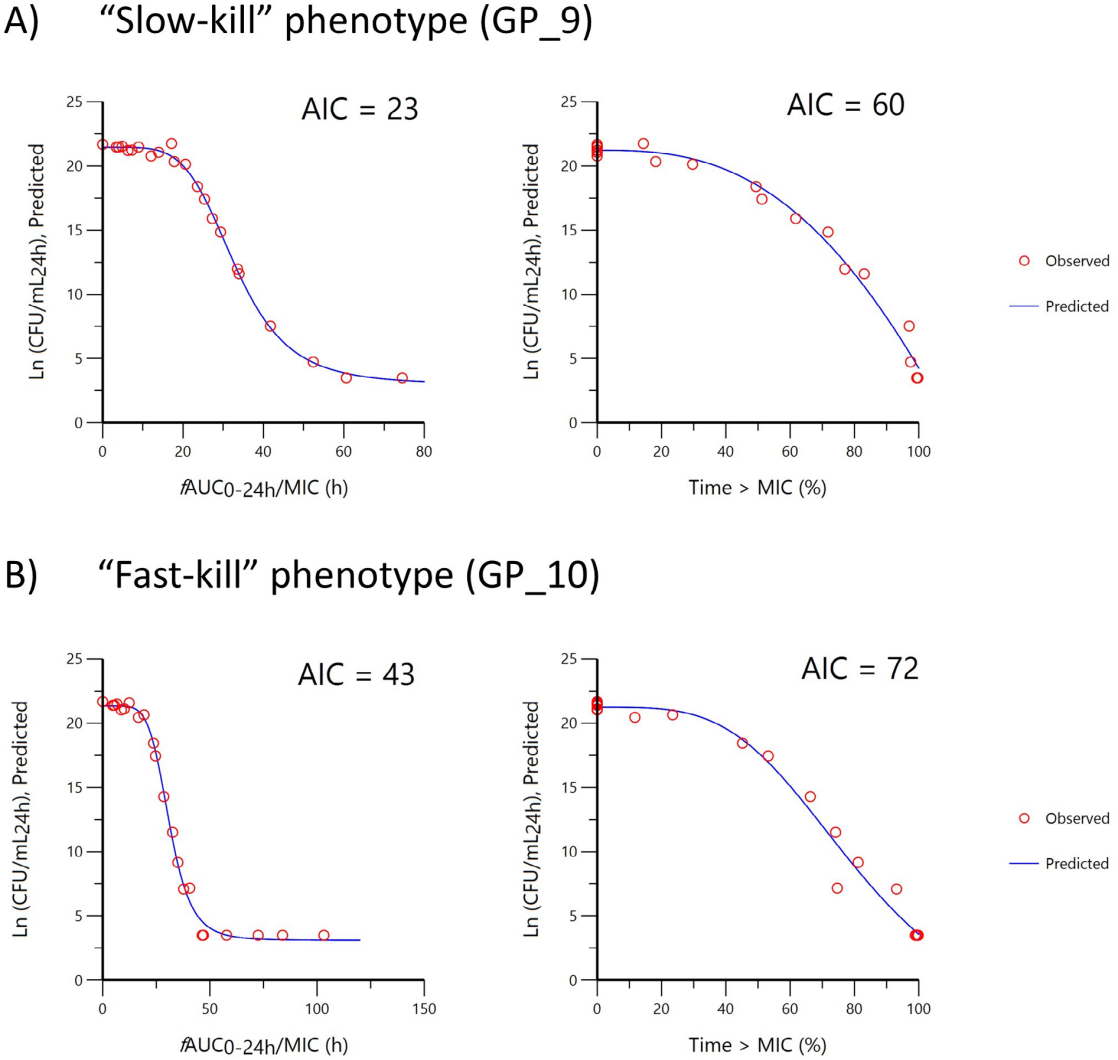
Comparison of fitting for the predicted natural log of bacterial density at 24 h (Ln CFU/mL_24h_) with *f*%T>MIC (percentage time Cfree exceeds predicted MIC in 24 h) or *f*AUC/MIC (area under the Cfree curve divided by predicted MIC over 24 h) for (A) *G. parasuis* “slow‐kill” (isolate GP_9), (B) “fast‐kill” (isolate GP_10). A sigmoid IMAX model was used. A lower AIC indicates a better model.

In silico dose fractionation using a combined IV PK/PD model with post hoc parameter values demonstrated that both “slow‐kill” and “Fast‐kill” isolates were more dependent on *f*AUC/MIC, than the percentage time Cfree exceeds predicted MIC in 24 h (Figure 7).

Bacteriostasis and 99.9% reduction from bacteriostasis were achieved for “slow‐kill” isolates when 24‐h average Cfree exceeded 1.46 × MIC (median, range: 1.39–1.51), and 1.63 × MIC (median, range: 1.53–1.69), respectively. For “fast‐kill” isolates these were achieved when 24‐h average Cfree exceeded 1.38 × MIC (median, range: 1.33–1.39), and 1.51 × MIC (median, range: 1.45–1.53), respectively (24‐h average Cfree calculated from *f*AUC/MIC_24_) as shown in Table 6.

**TABLE 6:** *f*AUC_24_/MIC to achieve bacteriostasis or a 99.9% (3log_10_) reduction from initial inoculum, for “slow‐kill” and “fast‐kill” isolates respectively.

Isolate	Posthoc estimate, *E*_max_ (h^−1^)	*f*AUC/MIC_24_ for bacteriostasis (h)	24‐h average Cfree/MIC for bacteriostasis (scalar)	*f*AUC/MIC_24_ for 99.9% reduction from stasis (h)	24‐h Average Cfree/MIC for 99.9% reduction from stasis (scalar)
“Slow‐kill” isolates					
GP_9	2.349	34.0	1.418	37.9	1.579
GP_3	2.558	33.4	1.393	36.8	1.534
GP_4	2.015	36.3	1.512	40.6	1.692
GP_6	2.056	35.9	1.495	40.4	1.685
“Fast‐kill” isolates					
GP_2	2.635	33.2	1.383	36.3	1.513
GP_5	2.735	33.3	1.387	36.6	1.526
GP_10	3.344	31.9	1.330	34.8	1.452

**FIGURE 8:**
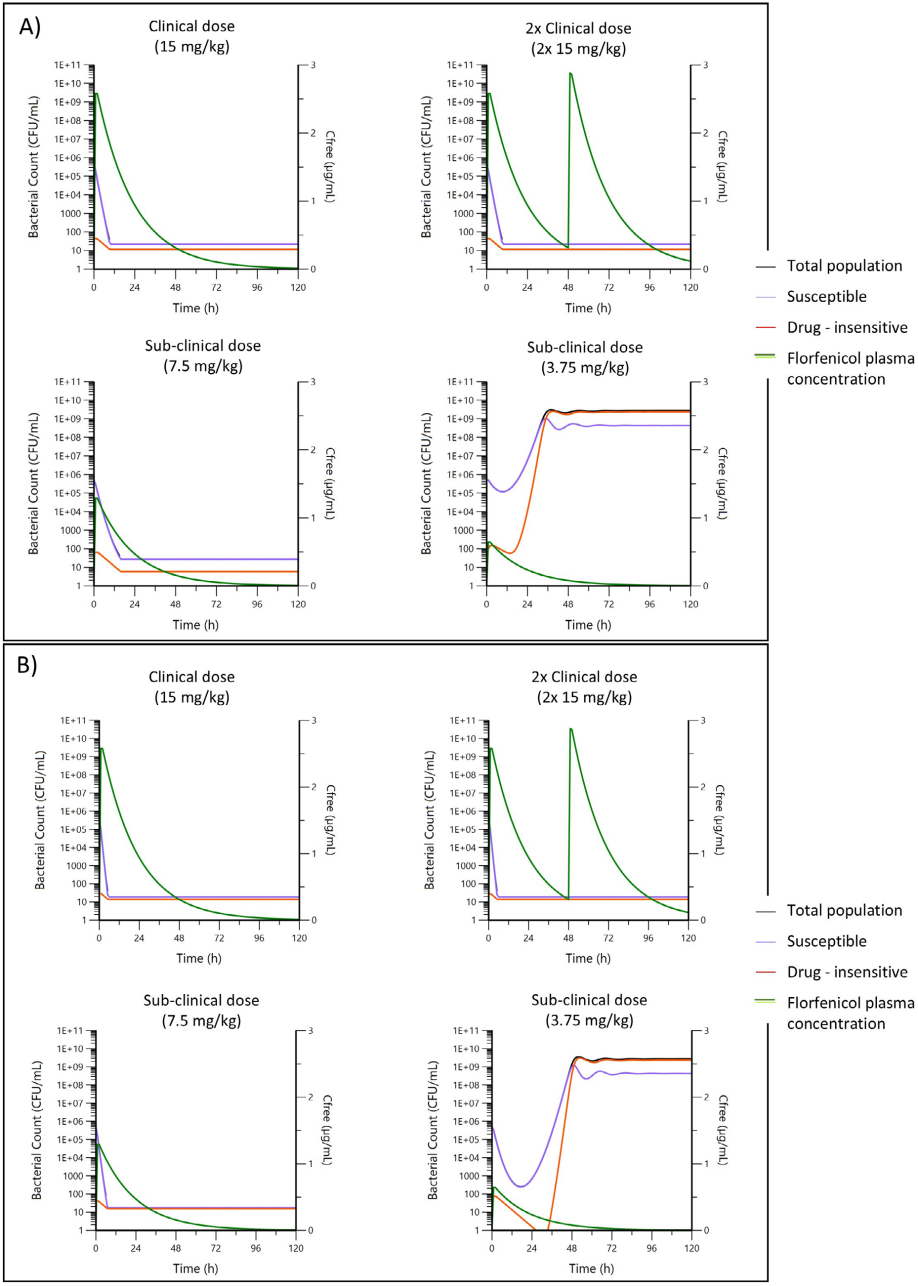
Simulated PK/PD profiles for a typical strain of (A) *G. parasuis* “slow‐kill” phenotype (GP_9) and (B) *G. parasuis* “fast‐kill” phenotype (GP_10) with an equivalent Nuflor formulation, free concentration (μg/mL; green line) adjusted for 15% protein binding, at the clinical dose once, a repeated clinical dose after 48 h, or at sub‐clinical half and quarter doses. Initial bacterial load of 5 × 10^5^ CFU/mL and predicted bacterial response for both the susceptible (blue line), persistent drug insensitive (red line), and total (black line) populations.

Clinical trial forecasting, for a 15 mg/kg standard formulation forecasted eradication of both “slow‐kill” (GP_9) and “fast‐kill” (GP_10) *G. parasuis* phenotypes (Figure 8). Furthermore, eradication may also be achievable at half the current clinical dose, although this would require further clinical testing.

## 4 Discussion

This study uses a PK/PD modeling approach across to comparatively assess the antibacterial effect of FFN against *G. parasuis*, *A. pleuropneumoniae* , and *P. multocida* , with a particular focus on differences in bacterial responses across isolates of *G. parasuis*. FFN, although considered to have a bacteriostatic mode of action has previously been characterised as bactericidal against various Pasteurellaceae species, including *P. multocida* and *Mannheimia haemolytica* (Sidhu et al. 2014; Blondeau, Shebelski, and Hesje 2015). The current study indicates the critical role concentration plays in the efficacy of FFN whilst also highlighting notable differences in the bactericidal kinetics of FFN between *G. parasuis* isolates, which is not evident in either *A. pleuropneumoniae* or *P. multocida*, a finding that has not been previously documented.

This consistent modelling approach enables a direct comparison of the pharmacodynamics of FFN between bacterial species, making it easier to understand species‐specific differences in drug efficacy and the optimal PK/PD indices for each pathogen. Such comparative modeling is crucial for improving dosing recommendations across different bacterial infections.

### 4.1 Florfenicol, *A. pleuropneumoniae* , and *P. multocida*

All three pathogens, *G. parasuis*, *A. pleuropneumoniae* , and *P. multocida* achieved similar bacterial densities during growth. However, an increased generation time was observed for *A. pleuropneumoniae* (31 min) and *P. multocida* (28 min), which has marginally faster doubling times than previously reported but still considered biologically plausible within this context (Bavananthasivam et al. 2012). This variation in growth rate could contribute to the differences in FFN's bactericidal kinetics observed among these bacterial species. Differences in experimental conditions, for example a pre‐incubation period to allow bacteria to acclimate, promoting optimal bacterial growth including a reduced or negligible lag‐phase and rapid logarithmic growth phase that may play an important role in the observed drug‐response kinetics. This may be a critical consideration when comparing studies. A previous study by Pelligand et al. (2019), time‐kill analysis of FFN against *P. multocida* showed delayed growth and required the inclusion of a “lag‐phase”, subsequently the determined PK/PD indices between that and the current study may be influenced by this difference and indicate that more experimental standardisation may be required for comparative studies.

The gamma Hill coefficient (gamma) varied moderately among the pathogens: *P. multocida* exhibited the lowest gamma, followed by *A. pleuropneumoniae* , while *G. parasuis* displayed the highest value. This indicates that the kill rate induced by FFN was less sensitive to concentration increment in *P. multocida* and *A. pleuropneumoniae* compared to *G. parasuis*. However, variation in gamma across isolates indicates overlap between *A. pleuropneumoniae* and *G. parasuis*. Additionally, differences in maximal efficacy (*E*_max_) were noted, with *A. pleuropneumoniae* showing the highest typical *E*_max_ (3.9 h^−1^), followed by *P. multocida* (3.2 h^−1^) and *G. parasuis* (2.5 h^−1^). Although there is some overlap in the confidence intervals, this trend suggests a possible species‐related variation in the maximal bacterial kill rate of FFN.

Comparative dose fractionation for *A. pleuropneumoniae* and *P. multocida* also indicated that *f*AUC/MIC was the best fit, showing that overall FFN is a more concentration‐dependent antibiotic and that this may be the most suitable PK/PD index across bacterial species. This aligns with previous studies that have also proposed that AUC/MIC may be the most suitable for *P. multocida* and *A. pleuropneumoniae* (Pelligand et al. 2019; Dorey et al. 2017; Illambas et al. 2013). The optimal PK/PD index depends on whether the antibiotic is time‐ or concentration‐dependent, with time‐dependent drugs favoring T>MIC and concentration‐dependent drugs favoring AUC/MIC, while FFN's mixed characteristics may cause variations in different models, and strain variation, and differences in the modelling approach (e.g., PK/PD index determined from integration over 24 h in this study compared to 96 h in the aforementioned studies) may influence the resulting PK/PD index.

### 4.2 Florfenicol and *G. Parasuis*

This study confirmed FFN's concentration‐dependent bactericidal effect against *G. parasuis*, classifying it as bactericidal at concentrations exceeding 2–4× MIC, consistent with other Pasteurellaceae species (Sidhu et al. 2014; Blondeau, Shebelski, and Hesje 2015). A novel finding, however, was the phenotypic variation in *E*_max_, or speed of kill, between *G. parasuis* isolates that shared identical MICs. This difference in bactericidal kinetics was consistent across multiple replicates, suggesting a true biological mechanism rather than stochastic variability. The “slow‐kill” and “fast‐kill” populations were statistically different for *E*_max_ (*p* = 0.04, 2‐sample *t*‐test), however statistically close to the significance threshold so interpretation should be considered with some caution, specifically it should be noted that this study was performed with a small number of isolates and a larger study should be performed to explore the full extent of biological variation. Importantly, the variation occurred in isolates without known FFN resistance genes, suggesting that intrinsic factors drive these differences.

One potential explanation for the observed variation in *E*_max_ could be differences in ribosomal properties or protein synthesis rates among the isolates. Previous research by Dickerman, Bandara, and Inzana (2020) identified two distinct clades within *G. parasuis* that exhibited differences in 16S rRNA sequences and gene copy numbers. These variations could support the lethal‐threshold hypothesis for ribosome inhibitors (Levin et al. 2017), which posits that a species‐specific threshold of incapacitated ribosomes must be reached for bactericidal activity. Further studies focusing on ribosomal sequences and the underlying mechanisms of ribosomal inhibitor‐induced cell death (Kohanski, Dwyer, and Collins 2010; Roberts et al. 2008; Baquero and Levin 2021) are necessary to fully elucidate this phenomenon.

The study demonstrated that the best‐fitted PK/PD index for FFN in *G. parasuis* was not influenced by the two described phenotypes, although it has been previously reported that PK/PD indices may differ between isolates not just between species (Gunderson et al. 2001), underscoring the importance of evaluating both time and concentration dependency. “Slow‐kill” and “fast‐kill” isolates aligned better with *f*AUC_24h_/MIC, a PK/PD index also used for *P. multocida* and *M. haemolytica* (Pelligand et al. 2019), however it is notable that the 24‐h average free concentration (relative to MIC) is lower in the “fast‐kill” phenotype. These findings emphasise the need for precise computation of target values for PK/PD indices to ensure effective clinical treatments.

### 4.3 Comparative Conclusions and Implications for Treatment

Although the target PK/PD indices are conserved across species, differences in the described pharmacodynamic targets should be considered, however they are unlikely to influence treatment decisions at the animal population level, they provide critical insight into dosing strategies. Since determining *E*_max_ is not feasible in routine clinical diagnostics, it is recommended to base treatment strategies on the “slow‐kill” phenotype, representing the worst‐case scenario. This strategy ensures that both phenotypic groups are effectively treated, as “slow‐kill” isolates require higher *f*AUC_24h_/MIC indices than “fast‐kill” isolates. The target PK/PD indices are reported here in terms of bacteriostasis and a 90% reduction from a static control (Lasko et al. 2022). An alternative is to consider a 90% or 99% reduction from a non‐static (i.e., growth) control (Rao et al. 2018) which results in target values less than those required to achieve stasis. Again, the target should be chosen based on the severity of the target infection type, and feasibility of achieving the target with clinical/non‐toxic doses at an expected range of wild‐type MICs (Bulitta et al. 2019).

Pharmacokinetic simulations of FFN demonstrated rapid bacterial eradication for both *G. parasuis* phenotypes, and for *A. pleuropneumoniae* and *P. multocida* supporting the use of current formulations for treating infections caused by these organisms, assuming that these isolates are representative of the broader bacterial population.

Further investigations are necessary to fully elucidate the molecular mechanisms driving these phenotypic differences. Whole‐genome sequencing, transcriptomics, and proteomics may help identify the factors contributing to the variations in FFN's bactericidal effects. In addition, a larger‐scale study involving more clinical isolates from diverse geographic regions would help to better understand the prevalence and clinical relevance of these phenotypes. Ultimately, while the results of this study strongly support the use of FFN for *G. parasuis* infections in the current formulation, future clinical trials should validate the in vitro findings with in vivo models, potentially informing regulatory decisions and improving the precision of treatment protocols.

## 5 Conclusion

In conclusion, this study offers a comprehensive characterisation of florfenicol's pharmacodynamic effects on *A. pleuropneumoniae*, *P. multocida*, and *G. parasuis*, confirming the findings from previous time‐kill studies that florfenicol's bactericidal activity is concentration dependent. For *G. parasuis*, the data also reveal an intriguing variation in kill rates between isolates with identical MICs. This finding underscores that bacteria of the same species, even with matching MICs, can exhibit different killing profiles, which could have implications for refining treatment strategies in the future. However, a larger study focused on *G. parasuis* should be conducted to better elucidate the extent of variation within the florfenicol PD of this species and to more comprehensively model the impact of florfenicol dosing. The pharmacokinetic simulations suggest that the currently used dosing regimens are likely appropriate for achieving effective bacterial eradication in *P. multocida* and *A. pleuropneumoniae* and that effectiveness would be achieved for *G. parasuis* even at a lower dose. Overall, these results highlight the robustness of florfenicol across major swine pathogens, while also calling attention to the need for a deeper understanding of intragenic variability in bacterial response to antimicrobial agents.

## Author Contributions

A.M., A.H. and L.P. contributed to conception and design of study. A.M., A.H., and S.A. organised and performed all aspects of the study. A.M., A.H., L.P., and P.L.T. performed the PD modelling analysis. A.M. and A.H. wrote the first draft of the manuscript. All authors contributed to the manuscript revision, read and approved the submitted version.

## Ethics Statement

This study did not involve the use of live animals. The pharmacokinetic (PK) data utilised were derived from previously published studies conducted in compliance with ethical standards and guidelines for the care and use of animals. These studies were approved by the appropriate institutional or national animal ethics committees, as required by the respective country's regulations.

## Conflicts of Interest

The funding for this study was provided by ECO Animal Health Ltd. AL (Eco Animal Health Ltd.) was involved in the study design, and revision of the submitted article. The funders were not involved in the collection, analysis, interpretation of data, the writing of this article, or the decision to submit it for publication. The remaining authors declare no conflicts of interest.

## Supporting information


Data S1.

## Data Availability

All data generated or analysed during this study are included in the manuscript and its Supporting Information. Additional datasets are available from the corresponding author upon reasonable request.

## Appendix A

**TABLE A1:** List of bacterial porcine isolates ( *A. pleuropneumoniae* , *G. parasuis*, and *P. multocida* ) received from ECO Animal Health on 29 July 2022.

Bacteria	Animal species	Date of sampling	Farm location	Age	Nature of the disease	Sample
*A. pleuropneumoniae* ‐6	Swine	20/03/2020	Huesca	Fattening	Respiratory	Lung
*A. pleuropneumoniae* ‐7	Swine	30/09/2020	Murcia	Fattening	Respiratory	Lung
*A. pleuropneumoniae* ‐8	Swine	10/02/2022	Zaragoza	Fattening (3 months)	Respiratory	Lung
*A. pleuropneumoniae* ‐9	Swine	06/04/2022	Badajoz	Fattening	Respiratory	Lung
*A. pleuropneumoniae* ‐10	Swine	12/04/2022	Navarra	Fattening (4 months)	Respiratory	Lung
*G. parasuis*‐2	Swine	19/02/2019	Lleida	—	—	Lung
*G. parasuis*‐3	Swine	22/10/2019	Teruel	Fattening	Respiratory	Lung
*G. parasuis*‐4	Swine	29/10/2019	Zaragoza	Weaning	Respiratory	Lung
*G. parasuis*‐5	Swine	28/11/2019	Ávila	Weaning	Respiratory	Lung
*G. parasuis*‐6	Swine	06/02/2019	Segovia	Weaning	Respiratory	Lung
*G. parasuis*‐9	Swine	29/01/2021	Zamora	Weaning 4 weeks	Respiratory	Lung
*G. parasuis*‐10	Swine	06/04/2022	Huesca	—	Respiratory	Lung
*P. multocida* ‐6	Swine	04/02/2020	Navarra	Weaning	Respiratory	Lung
*P. multocida* ‐7	Swine	12/06/2020	Ávila	—	Respiratory	Lung
*P. multocida* ‐8	Swine	25/02/2021	Castellón	Fattening	Respiratory	Lung
*P. multocida* ‐9	Swine	15/10/2021	Coruña, A	—	—	Lung
*P. multocida* ‐10	Swine	03/03/2022	Murcia	Fattening	Respiratory	Lung

## Appendix B Semi‐Mechanistic Model Description

The model describes bacterial density change as a growing drug‐sensitive population (sensitive, *S*, Equation 1) which transfers to a non‐replicating, drug‐insensitive population (*P*, Equation 2) at a rate *K*_SP_ (Equation 3) as the total population (*S* + *P*) reaches *B*_MAX_:

$$\frac{\mathrm{dS}}{\mathrm{dt}}=K_{\text{GROWTH}}\times S-K_{\text{DEATH}}\times S-K_{\mathrm{SP}}\times S+K_{\mathrm{SP}}\times P$$

$$\frac{\mathrm{dP}}{\mathrm{dt}}=K_{\mathrm{SP}}\times S-K_{\text{DEATH}}\times P-K_{\mathrm{SP}}\times P$$

$$K_{\mathrm{SP}}=\left(\frac{K_{\text{GROWTH}}-K_{\text{DEATH}}}{B_{\max}}\right)\times \left(S+P\right)$$

where *K*_GROWTH_ is the growth rate (replication rate), *K*_DEATH_ is natural death rate (constant, fixed to 0.179 h^−1^ (67)), B_MAX_ is the maximum bacterial density, *S* is the susceptible population at time (*t*), *P* is the non‐susceptible, non‐growing population at time (*t*). The total population was the primary experimental output, defined as the sum of *S* + *P* at each time point.

A sigmoidal Hill function (*E*_max_ model), relating the antimicrobial effect (*K*_DRUG_) to efficacy (*E*_max_), potency (EC_50_), and sensitivity (Gamma) is generally assumed to characterise the relationship between antimicrobial concentration and the effect on the bacterial population. Florfenicol effect was included as a concentration‐dependent killing rate (*K*_DRUG_) (Equation 4), additive to *K*_DEATH_ on the susceptible population only (Equation 5):

$$K_{\text{DRUG}\left(t\right)}=\frac{E_{\max}\times C{\left(t\right)}^{\text{Gamma}}}{{{\mathrm{EC}}_{50}}^{\text{Gamma}}+C{\left(t\right)}^{\text{Gamma}}}$$

$$\frac{\mathrm{dS}}{\mathrm{dt}}=K_{\text{GROWTH}}\times S-\left(K_{\text{DEATH}}+K_{\text{DRUG}}\right)\times S-K_{\mathrm{SP}}\times S+K_{\mathrm{SP}}\times P$$

where *E*_max_ (h^−1^) is the maximal killing rate (additional to natural death rate) for the susceptible population, EC_50_ is the florfenicol concentration (mg/L) in vitro to achieve 50% *E*_max_, and Gamma (a scalar) is the Hill coefficient (slope of concentration‐effect relationship). In vitro Florfenicol (free) concentrations were assumed to be static with no degradation over 24 h.

Individual estimation of EC_50_ was applied as an additive difference from the typical value of the reference isolate in the form:

$$\theta_{i}=\theta_{1}+{\Delta \theta }_{i}$$

where θ_1_ is the typical population value of the parameter, θ_i_ is the parameter value for the ith strain and Δθ_i_ is the deviation associated with the ith strain from the population value. Initial estimates for the deviation were given as the difference between observed MICs for each isolate and the typical WT strain (e.g., initial estimate of deviation in EC_50_ for GP_6 (MIC 0.5 mg/L) was +0.2 of the typical value for EC_50_ of GP_9 (MIC 0.3 mg/L)).

Modelling inter‐strain variability was evaluated by estimating the variance of model parameters (random effect). Random effects were introduced individually on parameters: EC_50_, *E*_max_, gamma, K_growth_, and B_max_ in the form:

$$\theta_{1i}=\theta_{1}\times \mathrm{Exp}\left(\eta_{1i}\right)$$

where θ_1_ is the typical population value of the parameter, θ_1i_ the value for parameter 1 during the ith TKC assay, and η_1i_ (eta) is the deviation associated with the ith strain from the corresponding population value. This exponential model assumes all parameters tested follow a log‐normal distribution.
