# Supplementary material for: Phenotype‐Specific Semi‐Mechanistic Modelling of Florfenicol Time‐Kill Curves in G. Parasuis Compared to Other Respiratory Pathogens
Source: J Vet Pharmacol Ther. 2025 Feb 7;48(4):318–39. doi: 10.1111/jvp.13500 (PMC12257270; doi:10.1111/jvp.13500)
Supplement: Supplementary file 1 — Data S1. [file JVP-48-318-s001.zip › jvp13500-sup-0001-FiguresS1-S7.docx]

**Supplementary Figure 1:** *A. pleuropneumoniae* isolate 6 (APP_6) time-kill curve analysis over a 24 h period at multiples of the MIC (0.40 mg/L) as determined by broth microdilution.

**Supplementary Figure 2:** *A. pleuropneumoniae* isolate 7 (APP_7) time-kill curve analysis (1^st^ replicate) over a 24 h period at multiples of the MIC (0.60 mg/L) as determined by broth microdilution.

**Supplementary Figure 3:** *A. pleuropneumoniae* isolate 7 (APP_7) time-kill curve analysis (2^nd^ replicate) over a 24 h period at multiples of the MIC (0.60 mg/L) as determined by broth microdilution.

**Supplementary Figure 4:** *A. pleuropneumoniae* isolate 7 (APP_7) time-kill curve analysis (3^rd^ replicate) over a 24 h period at multiples of the MIC (0.60 mg/L) as determined by broth microdilution.

**Supplementary Figure 5:** *A. pleuropneumoniae* isolate 8 (APP_8) time-kill curve analysis over a 24 h period at multiples of the MIC (0.60 mg/L) as determined by broth microdilution.

**Supplementary Figure 6:** *A. pleuropneumoniae* isolate 9 (APP_9) time-kill curve analysis over a 24 h period at multiples of the MIC (0.45 mg/L) as determined by broth microdilution.

**Supplementary Figure 7:** *A. pleuropneumoniae* isolate 10 (APP_10) time-kill curve analysis over a 24 h period at multiples of the MIC (0.60 mg/L) as determined by broth microdilution.
