# Supplementary material for: Phenotype‐Specific Semi‐Mechanistic Modelling of Florfenicol Time‐Kill Curves in G. Parasuis Compared to Other Respiratory Pathogens
Source: J Vet Pharmacol Ther. 2025 Feb 7;48(4):318–39. doi: 10.1111/jvp.13500 (PMC12257270; doi:10.1111/jvp.13500)
Supplement: Supplementary file 1 — Data S1. [file JVP-48-318-s001.zip › jvp13500-sup-0002-FiguresS1-S20.docx]

**Supplementary Figure 1:** *G. parasuis* isolate 2 (GP_2) time-kill curve analysis (1^st^ replicate) over a 24 h period at multiples of the MIC (0.25 mg/L) as determined by broth microdilution.

**Supplementary Figure 2:** *G. parasuis* isolate 2 (GP_2) time-kill curve analysis (2^nd^ replicate) over a 24 h period at multiples of the MIC (0.25 mg/L) as determined by broth microdilution.

**Supplementary Figure 3:** *G. parasuis* isolate 2 (GP_2) time-kill curve analysis (3^rd^ replicate) over a 24 h period at multiples of the MIC (0.25 mg/L) as determined by broth microdilution.

**Supplementary Figure 4:** *G. parasuis* isolate 3 (GP_3) time-kill curve analysis (1^st^ replicate) over a 24 h period at multiples of the MIC (0.3 mg/L) as determined by broth microdilution.

**Supplementary Figure 5:** *G. parasuis* isolate 3 (GP_3) time-kill curve analysis (2^nd^ replicate) over a 24 h period at multiples of the MIC (0.3 mg/L) as determined by broth microdilution.

**Supplementary Figure 6:** *G. parasuis* isolate 3 (GP_3) time-kill curve analysis (3^rd^ replicate) over a 24 h period at multiples of the MIC (0.3 mg/L) as determined by broth microdilution.

**Supplementary Figure 7:** *G. parasuis* isolate 4 (GP_4) time-kill curve analysis (1^st^ replicate) over a 24 h period at multiples of the MIC (0.25 mg/L) as determined by broth microdilution.

**Supplementary Figure 8:** *G. parasuis* isolate 4 (GP_4) time-kill curve analysis (2^nd^ replicate) over a 24 h period at multiples of the MIC (0.25 mg/L) as determined by broth microdilution.

**Supplementary Figure 9:** *G. parasuis* isolate 4 (GP_4) time-kill curve analysis (3^rd^ replicate) over a 24 h period at multiples of the MIC (0.25 mg/L) as determined by broth microdilution.

**Supplementary Figure 10:** *G. parasuis* isolate 5 (GP_5) time-kill curve analysis (1^st^ replicate) over a 24 h period at multiples of the MIC (0.4 mg/L) as determined by broth microdilution.

**Supplementary Figure 11:** *G. parasuis* isolate 5 (GP_5) time-kill curve analysis (2^nd^ replicate) over a 24 h period at multiples of the MIC (0.4 mg/L) as determined by broth microdilution.

**Supplementary Figure 12:** *G. parasuis* isolate 5 (GP_5) time-kill curve analysis (3^rd^ replicate) over a 24 h period at multiples of the MIC (0.4 mg/L) as determined by broth microdilution.

**Supplementary Figure 13:** *G. parasuis* isolate 6 (GP_6) time-kill curve analysis (1^st^ replicate) over a 24 h period at multiples of the MIC (0.5 mg/L) as determined by broth microdilution.

**Supplementary Figure 14:** *G. parasuis* isolate 6 (GP_6) time-kill curve analysis (2^nd^ replicate) over a 24 h period at multiples of the MIC (0.5 mg/L) as determined by broth microdilution.

**Supplementary Figure 15:** *G. parasuis* isolate 6 (GP_6) time-kill curve analysis (3^rd^ replicate) over a 24 h period at multiples of the MIC (0.5 mg/L) as determined by broth microdilution.

**Supplementary Figure 16:** *G. parasuis* isolate 9 (GP_9) time-kill curve analysis (1^st^ replicate) over a 24 h period at multiples of the MIC (0.3 mg/L) as determined by broth microdilution.

**Supplementary Figure 17:** *G. parasuis* isolate 9 (GP_9) time-kill curve analysis (2^nd^ replicate) over a 24 h period at multiples of the MIC (0.3 mg/L) as determined by broth microdilution.

**Supplementary Figure 18:** *G. parasuis* isolate 9 (GP_9) time-kill curve analysis (3^rd^ replicate) over a 24 h period at multiples of the MIC (0.3 mg/L) as determined by broth microdilution.

**Supplementary Figure 19:** *G. parasuis* isolate 10 (GP_10) time-kill curve analysis (1^st^ replicate) over a 24 h period at multiples of the MIC (0.3 mg/L) as determined by broth microdilution.

**Supplementary Figure 20:** *G. parasuis* isolate 10 (GP_10) time-kill curve analysis (2^nd^ replicate) over a 24 h period at multiples of the MIC (0.3 mg/L) as determined by broth microdilution.

**Supplementary Figure 21:** *G. parasuis* isolate 10 (GP_10) time-kill curve analysis (3^rd^ replicate) over a 24 h period at multiples of the MIC (0.3 mg/L) as determined by broth microdilution.
